# Clinical and Functional Characterization of URAT1 Variants

**DOI:** 10.1371/journal.pone.0028641

**Published:** 2011-12-16

**Authors:** Velibor Tasic, Ann Marie Hynes, Kenichiro Kitamura, Hae Il Cheong, Vladimir J. Lozanovski, Zoran Gucev, Promsuk Jutabha, Naohiko Anzai, John A. Sayer

**Affiliations:** 1 Medical School, University Children's Hospital, Skopje, Macedonia; 2 Institute of Genetic Medicine, Newcastle University, Central Parkway, Newcastle upon Tyne, United Kingdom; 3 Department of Nephrology, Kumamoto University Graduate School of Life Sciences, Kumamoto, Japan; 4 Department of Pediatrics, Seoul National University Children's Hospital, Seoul, Korea; 5 Department of Pharmacology and Toxicology, Dokkyo Medical University School of Medicine, Mibu, Tochigi, Japan; National Cancer Institute, United States of America

## Abstract

Idiopathic renal hypouricaemia is an inherited form of hypouricaemia, associated with abnormal renal handling of uric acid. There is excessive urinary wasting of uric acid resulting in hypouricaemia. Patients may be asymptomatic, but the persistent urinary abnormalities may manifest as renal stone disease, and hypouricaemia may manifest as exercise induced acute kidney injury. Here we have identified Macedonian and British patients with hypouricaemia, who presented with a variety of renal symptoms and signs including renal stone disease, hematuria, pyelonephritis and nephrocalcinosis. We have identified heterozygous missense mutations in *SLC22A12* encoding the urate transporter protein URAT1 and correlate these genetic findings with functional characterization. Urate handling was determined using uptake experiments in HEK293 cells. This data highlights the importance of the URAT1 renal urate transporter in determining serum urate concentrations and the clinical phenotypes, including nephrolithiasis, that should prompt the clinician to suspect an inherited form of renal hypouricaemia.

## Introduction

In man, the level of serum uric acid is determined primarily by the production of urate, as an end product of purine metabolism (for which the liver enzyme xanthine oxidase is necessary) versus biliary and urinary tract elimination. In the majority of other mammals, uric acid is metabolized by uricase (urate oxidase) to allantoin, before urinary excretion. Thus man (and other species lacking uricase, such as great apes), has comparably higher serum uric acid levels than most mammals.

The renal handling of uric acid is a complex and incompletely understood process [1], [2]. Uric acid is freely filtered at the glomerulus, the majority undergoes reabsorption via proximal tubular urate transporter proteins and a proportion (∼10%) is secreted back into the filtrate in the late proximal tubule. Molecular genetic and genome wide association studies have recently allowed the identification of several proximal tubule urate transporters including URAT1 (alias SLC22A12) [3] and GLUT9 (alias SLC2A9) [4], [5], [6]. Proposed models of urate transport in the proximal tubule [7] suggest an initial uptake of uric acid from the filtrate by URAT1, coupled to organic acid transporters. GLUT9, in two different isoforms, allows for basolateral exit of urate from the proximal tubule (isoform I) and regulation of urate entry/exit at the apical membrane (GLUT9ΔN isoform). Finally, in the late proximal tubule there are transporter proteins mediating uric acid secretion (including ABCG2, NPT1 and NPT4) [7]. As uric acid excretion is mediated through molecular transporters, certain drugs such as fenofibrate, valproic acid, trimethoprim and losartan may be used to manipulate these processes [8], [9], [10], thus allowing manipulation of serum uric acid levels.

In humans, genetic defects in the activity of xanthine oxidase or an acquired defect in liver enzyme function or renal uric acid handling may result in hypouricaemia. Acquired hypouricaemia may be seen in a number of clinical disorders, including Fanconi syndrome [11], type 1 and type 2 diabetes mellitus [12], [13], thyrotoxicosis [14], pseudohypoparathyroidism type 1b [15], pseudoaldosteronism due to licorice ingestion [16], distal renal tubular acidosis [17], [18], obstructive jaundice [19] and severe acute respiratory syndrome [20].

Idiopathic renal hypouricaemia is an inherited form of hypouricaemia that is characterized by excessive urinary wasting of uric acid leading to an increased clearance (and increased fractional excretion) of uric acid. The majority of patients are asymptomatic, but some may present with uric acid nephrolithiasis or acute kidney injury following severe exercise [21]. In 2002, Enomoto *et al.* reported that mutations in gene *SLC22A12* encoding the URAT1 transporter were responsible for most cases of idiopathic renal hypouricaemia [3]. Recently Anzai et al. found mutations in *SLC2A9*, encoding GLUT9, in patients with severe renal hypouricaemia [22]. It is noteworthy that reports of idiopathic renal hypouricaemia secondary to mutations in uric acid transporters URAT1 and GLUT9 were initially reported from Japan, Korea and China [23]. More recently, three Jewish Israeli families of Iraqi origin have been reported to have renal hypouricaemia, with a common mutation in *SLC22A12* [24]. Inactivating mutations in *SLC22A12* have not yet, to our knowledge, been reported in a Caucasian population.

The typical presentation of idiopathic renal hypouricaemia is that of exercise induced acute kidney injury with a preceding history of loin pain with nausea and vomiting for several hours after physical exercise. The exact mechanism of renal damage is unclear, but may relate to damage from oxygen free radicals [21]. In contrast to this dramatic presentation, most patients are well with no overt clinical symptoms, although renal stones and hematuria may be presenting symptoms and signs.

Here we present data from Skopje (Macedonia) and Newcastle upon Tyne (UK) where we have investigated the underlying genetic cause of hypouricaemia in patients of European descent. We present mutations in *SLC22A12* encoding URAT1 alongside their clinical, biochemical and functional characterization. This data highlights the importance of renal urate transporters in determining serum urate concentrations, and the clinical phenotypes that should lead the renal clinician to suspect an inherited form of renal hypouricaemia.

## Results

### Clinical descriptions

**Figure 1:**
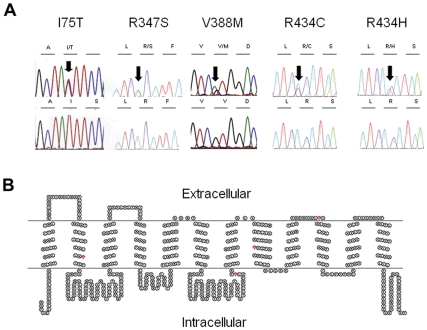
Identification of mutations within SLC22A12 encoding URAT1. A. Chromatograms showing sequence data and translated amino acids. These demonstrate heterozygous missense variants (above, arrowed) and normal controls (below). B. SLC22A12 encodes URAT1, a 553 amino acid protein with a predicted 12 Transmembrane domains (TMPred). It has an intracellular N- and C- terminus. Amino acid residues implicated in hypouricaemia and modelled in the present study are highlighted in red and include Isoleucine, I at position 75, arginine, R at position 347, Valine V at position 388 and Arginine, R at position 434.

A total of thirty two patients with hypouricaemia were recruited (Macedonia, n = 20 and United Kingdom n = 12) for mutational analysis of the *SLC22A12* and *SLC2A9* genes. The basic demographic, clinical, laboratory and molecular genetic data from these patients are given in Table 1. We found changes in *SLC22A12* in five patients from Macedonia and two patients from the United Kingdom (Figure 1A). No pathogenic mutations in *SLC2A9* were identified. An outline of the clinical, biochemical and molecular genetic features of each case is given below.

**Table 1:** Demographic, clinical, biochemical and molecular genetic data on 6 patients with renal hypouricaemia.

Patient	Age (yrs)	Sex	Serum Urate(mg/dl)	FE_urate_ (%)	Renal symptoms*	Comorbidity	URAT1 mutation leading to missense
SK-1	7	F	0.72	21	Nephrolithiasis	Cyclic vomiting	p.R434C
SK-2	18	F	1.75	27	Previous pyelonephritis	Reflux nephropathy; Hypertension	p.R434H
SK-3	5.5	F	1.24	31	Nephrocalcinosis	Distal renal tubular acidosis	p.R434C
SK-4	8	M	1.24	27	Recurrent episodes of gross hematuria and renal colic	Alport syndrome	p.R347S
SK-5	7	F	1.28	17	None	Hashimoto thyroiditis; vitiligo	p.R434H
NC-1	41	F	2.00 (transient)	16	Recurrent nephrolithiasis	Type I diabetes mellitus; hypothyroidism	p.V388M
NC-2	45	F	2.02	N/A	Recurrent nephrolithiasis	None	p.I75T

*Symptoms due to renal hypouricaemia, SK-Skopje, NC-Newcastle, FE_urate_- Fractional excretion of uric acid, N/A- not available.

Patient SK-1: This 7 year old girl was referred to the nephrology unit with persistent vomiting, presumed to be secondary to a non-obstructive kidney stone. She had a history of similar episodes during the preceding 6 months. On admission she was alert, but pale and moderately dehydrated. Clinical examination revealed that her abdomen was non-tender with no renal masses. A renal ultrasound scan (USS) revealed a single stone within the right kidney measuring 10 mm, without calyceal dilatation. Laboratory investigations on admission revealed normal renal function (serum creatinine 26 µmol/l, estimated glomerular filtration rate 123 ml/min/1.73 m^2^ (according to Schwartz formula)). Although she was moderately dehydrated, she had persistently low serum uric acid levels ranging from 0.72–1.18 mg/dl. FE_urate_ was elevated at 20.8%. Liver function tests, serum electrolytes, total protein and albumin were within reference values. Urinary excretion of cystine, calcium, phosphate, oxalate, glucose, low molecular weight proteins, amino acids and organic acids were also within reference limits. In order to clarify the etiology of urolithiasis, urinary excretion of xanthine and hypoxanthine was measured, showing normal values. Mutation analysis of *SLC22A12* revealed a heterozygous missense mutation, leading to amino acid change p.R434C. Genetic screening of other family members revealed that her brother and father both carried the identical heterozygous change, but were asymptomatic in terms of renal stones, with normal renal ultrasound scan and no reported episodes of exercise induced renal failure. Biochemical evaluation revealed that the father had normal values of serum uric acid while the brother had moderately decreased serum uric acid level (1.56 mg/dl).

Patient SK-2: This 18 year female was referred for management of hypertension. She had a past medical history of pyelonephritis and bilateral ureteric reflux with scarring of the left kidney, as determined by USS and Tc^99^DMSA scan. Laboratory investigations revealed serum creatinine and urea within normal limits. She was found to have low serum uric acid levels on two occasions (1.74 mg/dl and 1.78 mg/dl, respectively) with an increased FE_urate_. She had mild proteinuria (0.35 g/24 h) and a renal USS revealed a small left kidney, but no evidence of nephrolithiasis. Mutational analysis of the *SLC22A12* gene was performed and a heterozygous missense mutation p.R434H was detected.

Patient SK-3: This 5 year old girl was admitted to the nephrology unit with severe dehydration, polyuria and vomiting. She was noted to have faltering growth and rickets. Laboratory investigations revealed a hyperchloremic metabolic acidosis (pH 7.23, HCO_3_ 13.6 mmol/l, BE −12.6 mmol/l), hypokalemia (3.0 mmol/l), hypophosphatemia (0.84 mmol/l) and hypouricaemia (1.24 mg/dl). Simultaneous measurement of urine pH with electrode revealed value of 6.77 which pointed to distal acidification defect (expected urine pH<5.5). There was evidence of proteinuria on urine dipstix testing (1+) which was characterized with SDS-PAG electrophoresis as complete tubular proteinuria. There was a generalized hyperaminoaciduria, but no glucosuria. There was also evidence of uricosuria (FE_urate_ varied between 24–31%) and hyperphosphaturia (FE_PO4_ varied between 21–33%). Ultrasound examination revealed bilateral nephrocalcinosis and a solitary cyst in the left kidney measuring 10 mm. Following alkali therapy, metabolic compensation was achieved and her growth pattern improved. Following correction of the metabolic acidosis, the proximal defects of aminoaciduria and low molecular proteinuria resolved. Serum electrolytes also normalized, but there was persistent hypouricaemia during the two year observational period (1.24–1.36 mg/dl). Since the hypouricaemia was associated with an elevated FE_urate_, we undertook mutational analysis of *SLC22A12* which revealed a heterozygous missense mutation, leading to amino acid change R434C. The mother of patient SK-3 (R434C) was also heterozygous for the R434C variant in *SLC22A12*. Biochemical evaluation confirmed she had a low normal serum uric acid level (2.3 mg/dl) with fractional excretion of urate of 19.3%. She had a normal renal ultrasound, but a past medical history of renal colic and passage of a single calculus 10 years ago.

Patient SK-4: This 8 year old boy presented with recurrent attacks of visible hematuria and sensorineural hearing loss. A percutaneous renal biopsy was performed which demonstrated minimal glomerular abnormalities on light microscopy. Immunofluorescence studies did not reveal any immune deposits and electron microscopic analysis was not available. Given a clinical suspicion of Alport syndrome, genetic studies were performed, identifying a mutation in the *COL4A5* gene. Following another attack of visible hematuria with very severe colicky pain, serum biochemistry revealed a low uric acid level. Repeated examinations of the uric acid confirmed persistent hypouricaemia, with high FE_urate_. Mutational analysis of *SLC22A12* revealed a heterozygous missense mutation, leading to the amino acid change R347S. Genetic analysis confirmed that the mother of patient SK-4 was also heterozygous for the sequence variant R347S.

Patient SK-5: A 7 year old female presented with vitiligo, prompting a screen for autoimmune diseases. She was found to have compensated hypothyroidism, with typical ultrasound changes of the thyroid gland and increased antithyroid antibodies, suggesting Hashimoto thyroiditis. She was also found to have hypouricaemia with significant hyperuricosuria. She underwent mutation analysis of *SLC22A12* which revealed a heterozygous missense mutation, p.R434H.

Patient NC-1: A 41 year old female presented with recurrent renal colic, with 2 episodes in less than 12 months. She received lithotripsy treatment for a left renal calculus (struvite stone). Her past medical history was noteworthy for type 1 diabetes mellitus, since 10 years of age, complicated by peripheral neuropathy and urinary tract infections. She also had treated hypothyroidism. At presentation, serum electrolyte abnormalities revealed a borderline low uric acid level and a serum creatinine of 95 µmol/L. A FE_urate_ was transiently raised at 16%. Repeat serum biochemistry revealed a serum uric acid level of 3.86 mg/dl and a normalised FE_urate_. Following successful lithotripsy she has remained stone free for 5 years, following advice regarding increased fluid intake. Genetic analysis of *SLC22A12* revealed a heterozygous missense mutation, p.V388M.

Patient NC-2: A 45 year old lady presented to the regional lithotripsy unit for treatment. She had recurrent renal stone disease (calcium oxalate) for 7 years, requiring both lithotripsy and a right pyeloplasty. She was on no medications. Serum electrolytes revealed a normal serum creatinine (75 µmol/l) with a low serum uric acid (2.01mg/dl). FE_urate_ was not available for this patient. We performed genetic analysis of *SLC22A12*, which revealed a heterozygous missense mutation, p.I75T.

### In silico analysis of mutations

Following the identification of variants within the *SLC22A12* gene encoding URAT1 (Figure 1A,B) we used online databases and mutation prediction software to attempt to score the pathogenicity of each variant. Within the URAT1 amino acid sequence, residues R347, V388 and R434 are highly conserved, whilst I75 is not well conserved (Table 2). SIFT and Snps3d analysis predicted all 5 sequence variants to be either not tolerated (SIFT) or deleterious (Snps3D). Polyphen analysis predicted that 3 out of the 5 missense changes to be “probably damaging”, with V388M and I75T predicted to be benign (Table 2).

**Table 2:** URAT1 variants and their *in silico* analysis for conservation and pathogenicity.

Missense change in URAT1	Evolutionary conservation	SNP ID (Average Hetrozygosity Index)	SIFT analysis	Snps3d analysis	PolyPhen-2 analysis
**I75T**	Not conserved between human, mouse and zebrafish	rs141570522 (0.001)	Predict Not Tolerated	Deleterious	Predicted to be benign
**R347S**	Conserved Human to Mouse	Novel variant	Predict Not Tolerated	Deleterious	Predicted to be probably damaging
**V388M**	Conserved between human, mouse and zebrafish	rs146388519 (0.000)	Predict Not Tolerated	Deleterious	Predicted to be benign
**R434C**	Conserved between human, mouse and zebrafish	rs145200251 (0.001)	Predict Not Tolerated	Deleterious	Predicted to be probably damaging
**R434H**	Conserved between human, mouse and zebrafish	rs147647315 (0.011)	Predict Not Tolerated	Deleterious	Predicted to be probably damaging

### Functional studies of URAT1 variants

Using mammalian cells, we evaluated the urate transport function of URAT1 sequence variants found in this study. Urate uptake was measured in transiently transfected HEK293 cells, comparing wild-type transporter activity to sequence variants I75T, R347S, R434C and R434H.

**Figure 2:**
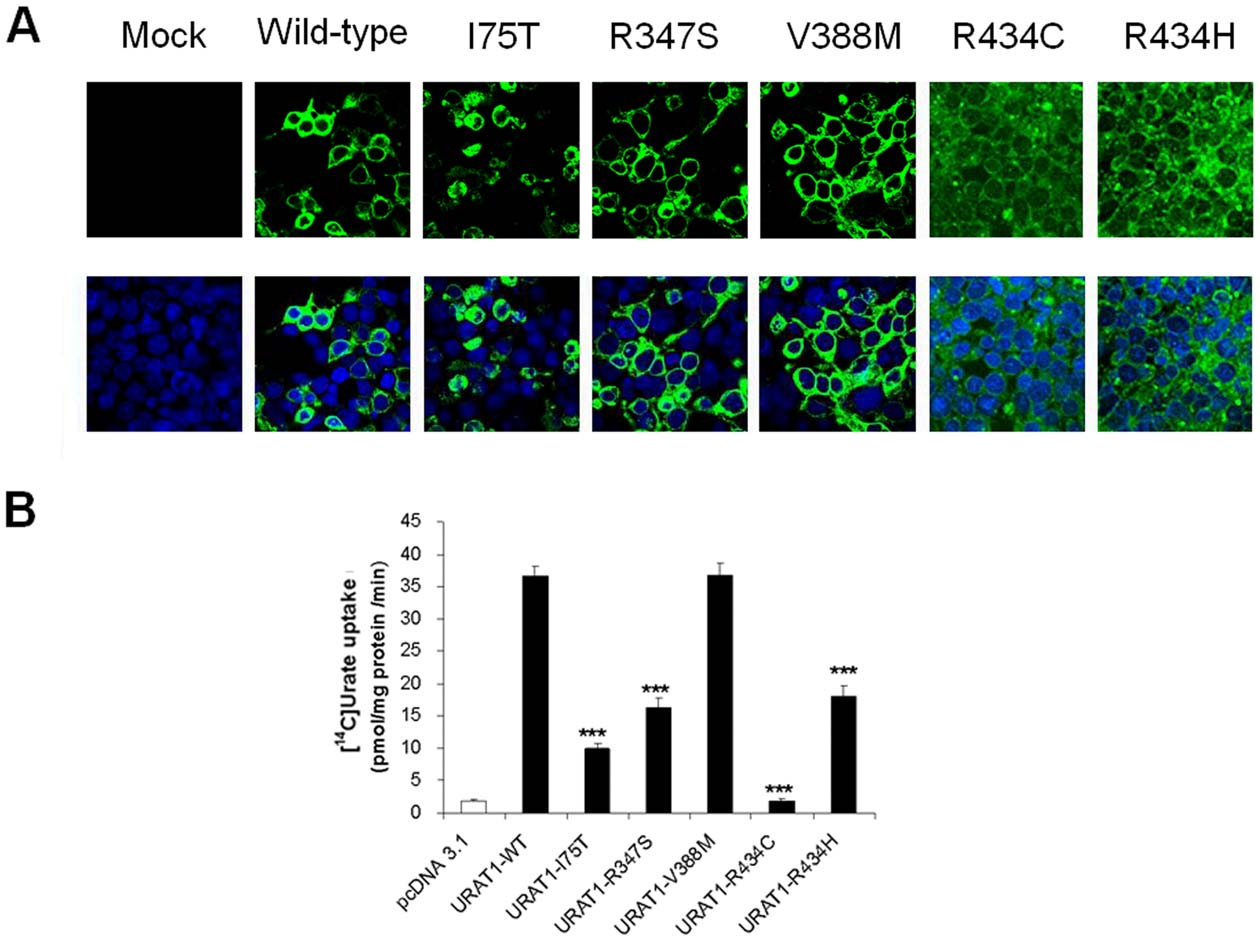
Functional analysis of changes in URAT1 expressed in HEK293 cells. A. HEK293 cells were transiently transfected with Flag-tagged URAT1 cDNA constructs (wild-type and variants I75T, R347S, V388M, R434C and R434H). Plasma membrane expression was detected using an anti-FLAG monoclonal antibody, secondarily detected using an Alexa Fluor® 488 (green) antibody. Nuclei are counter stained using DAPI (blue). B. Uric acid uptake by HEK293 cells transiently transfected with wild-type URAT1 or its mutants was measured using [^14^C]Urate at 2 min, at 37°C and pH7.4. Significant reductions in urate transport activity was seen in some of the disease-associated variants. Data are mean ± S.E.M with n = 4. ***, P<0.001 when compared with wild type.

Following transient transfection of HEK293 cells with FLAG-tagged URAT1 constructs, we demonstrate strong plasma membrane expression in wild-type URAT1 (Figure 2A).

Plasma membrane expression levels of variants R434C and R434H were low whereas the intracellular localization was not strongly observed, possibly due to the stability of protein. For variant I75T the partial membrane expression was observed together with partial intracellular localization. For variants R347S and V388M strong plasma membrane expression, similar to wild-type levels was observed (Figure 2A).

Measurement of urate uptake in HEK293 cells demonstrates a significant reduction of urate transport function by I75T, R347S, R434C and R434H variants of URAT1 but not in V388M variant (Figure 2B).

## Discussion

Idiopathic renal hypouricaemia is a disorder that has been characterized previously in patients from Far Eastern countries including Japan, Korea and China. The disease may be completely asymptomatic [23], [25], [26], but there are many reports, particularly from Japan, where patients present with acute kidney injury following strenuous physical activities [21], [27], [28], [29], [30], [31], [32], [33], [34]. The exact mechanism of acute kidney injury remains elusive, but it is believed that uric acid serves as an antioxidant, and in states of hypouricaemia this protective role is lost. Kaneko et al. demonstrated in a 15 year old girl with idiopathic renal hypouricaemia an oxidative imbalance soon after exercise with a predisposition to exercise-induced acute renal failure [35]. In contrast, some patients may present with more minor renal features including nephrolithiasis or hematuria [23], [31], [36].

In 2002, Enomoto et al. established that URAT1 transporter was responsible for tubular reabsorption of urate [3]. *SLC22A12* encodes the protein URAT1 and loss of function mutations are responsible for majority of patients with idiopathic renal hypouricaemia. The W258X variant of URAT1 is a typical mutation found in Japanese and Korean populations [23], [31], [37], [38]. Allele frequency of W258X in the general population in Japan was found to be as high as 1.9% [39]. Heterozygous carriers of URAT1 mutations are usually asymptomatic but they may develop nephrolithiasis. Prevalence of hypouricaemia in Japan varies between 0.15% [40] and 0.23% from the analysis of serum urate levels in 1730 school children [41]. In Korea, the prevalence of hypouricaemia in healthy adults is 3.3% [42]. Therefore, school-age children who plan to performing competitive sporting activities are advised to have their serum uric acid level checked [43].

Loss of function mutations affecting URAT1 have not been previously reported in a Caucasian population. However, single case reports from European patients presenting with clinical and biochemical features of hereditary renal hypouricaemia exist [44], [45], [46], [47]. Tzovaras et al. tested nine Greek subjects with primary renal hypouricaemia [48]. All had serum uric acid levels <2.5 mg/dl, associated with a FE_urate_>10% and no other known cause of hypouricaemia. No definite pathogenic mutations were detected in this series and just one silent polymorphism (1246T>C) in exon 2 of the *SLC22A12* gene was noted. It is questionable why hereditary renal hypouricaemia is apparently so rare in Caucasian populations. Either the prevalence of URAT1 mutations is indeed low in this population, or a decreased awareness of this disease and its presentation outside of the Far East allows cases to go undetected. Even in Japan, there are patients who have clinical features of renal hypouricaemia but no *SLC22A12* mutations. Very recently, a genome-wide association study was performed in 6890 African Americans and 21708 European participants in order to try and identify risk alleles for elevated serum urate, associated with gout [49]. A novel URAT1 variant (G65W) was identified (rs12800450) and was associated with a reduction in serum urate of around 1.2 mg/dl per copy of the minor allele. Urate transport studies demonstrated a reduction in the urate transport for the G65W URAT1 variant [49]. This study validates heterozygous changes within URAT1 as a determinate of reduced serum urate levels. In this study the variant allele provided a protective affect against gout, but one may postulate that this may also be an at-risk allele for the hypouricaemia. Both biochemically and clinically, a single heterozygous change in URAT1 may be significant. Cheong et al. reported a W258X homozygous mutation in a 7 year old child, whose mother and brother were also heterozygous for W258X and had mild hypouricaemia and abnormally high FE_urate_, whilst his father who was also heterozygous for W258X, had a normal serum uric acid level of 4.6 mg/dL [23]. In the same report, an 11-year-old girl with asymptomatic microscopic hematuria and low serum urate was a compound heterozygote for W258X/R477H. Her mother had a heterozygous W258X mutation associated with renal hypouricaemia (serum uric acid 2.3 mg/dl and FE_urate_ 16.3%) [23]. In addition, a 15 year old girl with haematuria, proteinuria and renal hypouricaemia (serum uric acid 1.3 mg/dl and FE_urate_ 14.5%) was noted to have a single heterozygous W258X mutation, whilst a 36-year-old female, with recurrent episodes of uric acid stones in the right ureter had a serum uric acid of 1.8 mg/dL and an elevated FE_urate_ of 28.1%. She was heterozygote for a R90H variant in URAT1 [23]. Vázquez-Mellado et al. reported patients heterozygous for C850G in URAT1 with primary gout and low serum uric acid concentrations [50] whilst Ichida, et al. reported 5 individuals with a W258X heterozygous change, one of whom had a history of acute kidney injury and renal stones [23], [31], [36].

Recently, the GLUT9 glucose transporter, encoded by *SLC2A9* gene has been shown to have an important functional role in transporting uric acid from the renal tubular cells through the basolateral membrane into interstitium [22]. Two additional reports have confirmed this finding [51], [52]. Matsuo et al. detected two heterozygous mutations in GLUT9 in two hypouricaemic subjects whom were negative for URAT1 mutations (R380W in exon 10 and R198C in exon 6) and confirmed their reduced transport activity in *Xenopus* oocyte expression system [51] whilst Dinour et al. recently described two families with recessively inherited hypouricaemia who were negative for URAT1 mutations [52]. Among hypouricaemic subjects in both families, three subjects had nephrolithiasis and three subjects had a history of exercise induced acute kidney injury. With genome wide homozygosity screen and linkage analyses they established *SLC2A9* as a causative gene for renal hypouricaemia in these families. In these families, homozygous carriers of *SLC2A9* mutations had very low serum levels of uric acid and extremely high values of FE_urate_ (at around 150%).

In this report we present combined data from Macedonian and English patients who are heterozygous carriers of *SLC22A12* sequence variants. Although the presentations of the patients were varied, a systematic search for hypouricaemia identified patients with possible hereditary renal hypouricaemia and who are suitable for mutational analysis of *SLC22A12* and *SLC2A9* genes to try and identify abnormalities in the encoded urate transporters URAT1 and GLUT9, respectively. We identified seven patients harboring five *SLC22A12* variants. Interestingly, three Macedonian patients carried mutations at amino acid position 434 of URAT1.

Some of these variants in *SLC22A12* were discovered following significant clinical episodes including nephrolithiasis in patients SK-1, NC-1 and NC-2. There were however, no episodes of exercise induced acute kidney injury that we are aware of.

The sequence variant R434H was identified in one of our patients with hypouricaemia. This variant has a reported heterozygosity index of 0.011 (Table 2), although this variant was not detected in our normal control subjects. Additional studies are required to demonstrate whether this variant is a common cause of hypouricaemia.

Of note, in our cohort of patients, we found no novel sequence variants in the GLUT9 transporter (*SLC2A9*), leaving a number of patients with unexplained hypouricaemia. However, given the complexity of proximal tubule urate handling, other urate transporter protein variants may account for hypouricaemia in the remaining patients.

In patient SK-1 nephrolithiasis was likely to be due to multiple risk factors including renal wasting of urate, episodes of cyclic vomiting leading to concentrated urine and hypocitraturia. In patient SK-4, who also had a molecular genetic diagnosis of Alport syndrome, attacks of gross hematuria were confusing due to presence of colicky pain and eumorphic red blood cells (100%), which are not usual features of Alport syndrome. In this patient, we found the mutation p.R347S. Cheong et al. reported a similar case to this, where a 14 year old girl who presented with acute post-streptococcal glomerulonephritis [23]. Although her nephritis had a favorable course, the microhematuria persisted more than one year. On reevaluation this girl was found to have low serum uric acid (1.3 mg/dl) with increased FE_urate_ (14.5%) in favor of idiopathic hypouricaemia. Mutational analysis in this patient revealed heterozygous W258X mutation in *SLC22A12*.

Patient SK-2 has hypertension and moderate proteinuria due to reflux nephropathy, presumably as a coincidental finding to the functionally significant p.R434H variant. As hypertension in the context of renal disease is often treated with ACE inhibitors or angiotensin receptor antagonists, some caution is advisable. Both losartan [8] and irbesartan [53] have an inhibitory action on URAT1. Thus treatment with these agents has potential to have a marked uricosuric effect in patients with homozygous URAT1 mutations.

Patient SK-3 had a complex phenotype of distal renal tubular acidosis and renal hypouricaemia, associated with the p.R434C mutation within URAT1. It is well known that hypouricaemia may be associated with distal renal tubular acidosis at diagnosis [17], [18] as a part of transitory proximal tubular dysfunction. In additional, pharmacological agents may also disrupt proximal tubular handing of urate. In our case, hypouricaemia persisted for more than 2 years despite a normalization of other proximal tubular functions. Definitively, mutational analysis of *SLC22A12* with a functionally significant change (p.R434C) explained the persistent hypouricaemia in this patient.

Patient NC-1 was a recurrent calcium stone former, with a past medical history of type 1 diabetes. Here, serum urate levels were only borderline low and the FE_urate_ was also only transiently raised at 16%. The missense mutation p.V388M was associated with negative functional data, with no significant change in urate transport in HEK293 cell experiments. Inclusion of this case is helpful as the negative functional data and the transient hypouricaemia are consistent with this variant being benign. The dataset derived from the V388M variant allow a comparison to be made between it and the more functionally significant variants, acting as another negative control. Given the hypouricaemia was transient in this case, we do not assume that this sequence variant is causative. From our sequence variants of URAT1, mutations with an impact upon uric acid handling (when modeled in HEK293 cells) are associated with a persistent hypouricaemia. Patient NC-2 was also a recurrent stone former, with persistently low serum uric acid. The heterozygous missense mutation, p.I75T was confirmed to be functionally significant in HEK293 urate uptake studies.

In previously reported Japanese and Korean cases of idiopathic renal hypouricaemia secondary to *SLC22A12* mutations, the majority of patients have been shown to have either homozygous mutations or compound heterozygote mutations [23]. In our Caucasian population, disease associations have been made with single heterozygous changes in URAT1, suggesting that such a change is sufficient and that a dominant pattern of inheritance may be present.

In conclusion, we have identified Macedonian and British patients with hypouricaemia, who presented with symptoms including renal stone disease and haematuria. We have identified missense mutations in *SLC22A12* encoding URAT1. This data highlights the importance of renal urate transporters in determining serum urate concentrations and the of clinical phenotypes that should lead the clinician to suspect an inherited form of renal hypouricaemia.

## Materials and Methods

### Ethics Statement

For Macedonia patients, the Institutional Review Board and the Ethics Committee at the Medical Faculty and The Ministry of Education and Science, Skopje, Republic of Macedonia approved the study. Informed written consent was obtained by participants' parents or legal guardians. For UK patients, full ethical approval was obtained by the Newcastle and North Tyneside Research Ethics Committee and informed written consent were obtained from participants.

### Recruitment of patients

Macedonian patients: Children attending in-patient or out-patient nephrology service at the University Children's Hospital, Skopje were screened for hypouricaemia. Serum uric acid level was determined in children with contraction or expansion of the extracellular volume, acute kidney injury, chronic kidney disease, nephrolithiasis, tubular disorders, those receiving diuretics or nephrotoxic drugs, liver diseases, diabetes mellitus and thyroid diseases. Children less than two years of age were excluded from this study since they have physiologically lower levels of uric acid due to immaturity of the liver and renal tubular function. Where serum uric acid levels were <2 mg/dl and Fractional Excretion of urate (FE_urate_) was >10% (normal range 2–8%) and these biochemical findings persisted despite clinical improvement and normalization of other biochemical indices, or if no explanation was found for lower serum levels of uric acid, than idiopathic renal hypouricaemia was considered to be a likely diagnosis [23]. Patients with suspected renal hypouricaemia underwent additional studies, including ultrasound imaging of the urinary tract (if previously not performed) and molecular analysis of *SLC22A12* and *SLC2A9*.

United Kingdom Patients: Adult kidney stone formers attending for lithotripsy at the Newcastle upon Tyne NHS Foundation Trust Hospital, UK were recruited (following informed consent) and screened for serum and urinary biochemical abnormalities. Twelve patients with hypouricaemia (<2.6 mg/dl) underwent molecular analysis of *SLC22A12* and *SLC2A9*.

### Molecular analysis

Mutational analysis of *SLC22A12* and *SLC2A9* genes was performed using exon PCR and direct sequencing. (Oligonucleotide primer sequences are listed in Table S1). For control patient DNA screening, 92 samples were obtained from blood donor (healthy control) panels.

### In silico analysis of mutations

Online in silico analyses were performed when sequence variants were identified. These included SIFT (http://sift.jcvi.org/), PolyPhen (http://genetics.bwh.harvard.edu/cgi-bin/ggi/ggi.cgi) and SNPS3D (www.snps3d.org/).

### Functional expression studies

Full-length cDNA of human URAT1 was obtained as described previously [3] and tagged with 3× FLAG at the N-terminus. The QuickChange Site-Directed Mutagenesis Kit (Stratagene, La Jolla, CA) was used to introduce point mutations into URAT1 cDNA in the expression vector according to the instructions. Complementary oligonucleotides used for mutagenesis are described in Table S2. All the final cDNA sequences were confirmed by DNA sequencing.

For the evaluation of transport function of *SLC22A12* mutations, we used a mammalian cell expression system as described previously [54]. Briefly, HEK293 cells [55] were maintained in Dulbecco's modified Eagle's medium (DMEM) supplemented with 10% fetal bovine serum (FBS), 1 mM sodium pyruvate, 100 U/ml penicillin, and 100 mg/ml streptomycin (Invitrogen, Carlsbad, CA) at 37°C and 5% CO_2_. Transient transfection with Lipofectamine 2000 (Invitrogen) was performed according to the manufacturer's instructions. After transfection, the cells were grown 36–48 h before the experiments.

Cellular uptake of [^14^C]UA were measured in hURAT1 (or its mutants)-transfected HEK293 cells [55] grown on poly-D-lysine-coated 24-well plates. The cells were incubated in chloride-free Hanks' balanced salt solution (HBSS) containing the following in mM: 125 Na gluconate, 4.8 K gluconate, 1.2 KH_2_PO_4_, 1.2 MgSO_4_, 1.3 Ca gluconate, 5.6 glucose and 25 HEPES, pH 7.4) for 10 min. The uptake study was started by adding HBSS containing [^14^C]UA or to the plate. After 2 min, the cells were washed three times in ice-cold HBSS, then lysed in 0.1 M NaOH for 20 min followed by measurement of radioactivity by scintillation counting. The experiments were performed in quadruplicate per experiment and repeated twice. All the data are given as the mean ± S.D. The Student's *t*-test was used to determine significant differences. A value of *P*<0.05 was considered to be significant.

Immunocytochemical analyses were performed as previously described [56]. URAT1 (or its mutants)-transfected HEK293 cells were fixed with methanol and incubated with the anti-FLAG antibody (1∶50) (Sigma) followed by Alexa Fluor® 488-labelled goat anti-rabbit immunoglobulin (Invitrogen; diluted 1∶200). The staining was observed under a confocal laser scanning microscope (Fluoview FV1000, Olympus). Alexa 488 fluorescence was excited by Argon laser light of wavelength 488 nm.

## Supporting Information

Table S1 Oligonucleotide primers for exon PCR of SLC22A12 and SLC2A9.
(DOC)

Table S2 Oligonucleotide primer pairs used for site-directed mutagenesis of SLC22A12.
(DOC)
