# Supplementary material for: Clinical and Functional Characterization of URAT1 Variants
Source: PLoS One. 2011 Dec 16;6(12):e28641. doi: 10.1371/journal.pone.0028641 (PMC3241677; doi:10.1371/journal.pone.0028641)
Supplement: Table S1 — Oligonucleotide primers for exon PCR of SLC22A12 and SLC2A9. (DOC) [file pone.0028641.s001.doc]

## Table S1: Oligonucleotide primers for exon PCR of SLC22A12 and SLC2A9

| SLC22A12 |  |  |
| --- | --- | --- |
| Exon 1 | 5’-CAAGTGACACCAGCAGGCTGAT-3’ | 5’-ACCTTTGAGTCTGACCCACCGT-3’ |
| Exon 2 | 5’-TCACTGTTCCACAGGGTCTTGC-3’ | 5’-TCGAGGTTCAGAGAAGGTTCCA-3’ |
| Exon 3&4 | 5’-CCGCCTCAGCTCAGCGGGCAAGCAT-3’ | 5’-CCCCCGGGTGGAGAGTGGGCAGGAT-3’ |
| Exon 5 | 5’-GTACAGGGTAGCAGTCTGAGGCTGG-3’ | 5’-GCACCTCCTCCTCTCTGAGACCTTC-3’ |
| Exon 6 | 5’-CTGAGGTAAGGCTGGGTCCTCCTC-3’ | 5’-GTCTCCTGTCGGGAGTGACTGACA-3’ |
| Exon 7* | 5’-GCCAAACCCAAAGGGAAGCCATGCTGGCAAGG-3’ | 5’GCCACACCCACAATCTGCTCCACGCTCAGACAC-3’ |
| Exon 7nested | 5’-CCTGAGCCCCCACCGCCCATTGTT-3’ | 5’-CCTGCTCTAGTCCAGCACCTCCAA-3’ |
| Exon 8&9 | 5’-GCTGAAGGGAGCCCTCATCTGATCT-3’ | 5’-AGGCTGCCCTGTGCTAGGGTTCTC-3’ |
| Exon 10 | 5'-GGCCAAAGGGAGAAGAGTGGATAG-3’ | 5’-GTCTCTTCCTCTGACCGTCCCATC-3’ |
| *Exon 7 is amplified by two-steps of nested PCR |  |  |
| SLC2A9 |  |  |
| Putative promoter isoform 1 | 5’-GAAGGGAAGACTGTTCTTGG-3’ | 5’-CTAGTATCAGAAGCCTGGAG-3’ |
| Putative promoter isoform 2 | 5’-GGGATGGAAGATTTTTGAGC-3’ | 5’-ACTCAGCCAACAGGAACTGAGTG-3’ |
| Exon 1a | 5’-TTCTTTGCTTGGCATTACCC-3’ | 5’-GCCTTGGCATTCAACATTC-3’ |
| Exon 2 | 5’-GCAAACCTTTCGAAGACTGC-3’ | 5’-TTAACAGCCTCCCACTGACC-3’ |
| Exon 1b | 5’-TCAAATTGCCACACTTCCTG-3’ | 5’-GCCTGCCTTCCCCACAG-3’ |
| Exon 3 | 5’-GGACAAAGACTTCTCCTCCG-3’ | 5’-CGCACCCGAAGGTTTCC-3’ |
| Exon 4 | 5’-TTGTTTTGTACACTGGCTTGC-3’ | 5’-GGACCCTGACAATGACACAG-3’ |
| Exon 5 | 5’-CTGGGATGGACAGTTCAGTG-3’ | 5’-CTCACATTTTGGGACACCC-3’ |
| Exon 6 | 5’-GTGCTATTTAATGTCCAGGGC-3’ | 5’-GCAAAATACAGGGACCAGCTT-3’ |
| Exon 7 | 5’-CAGTCCCCTCAACAATGACC-3’ | 5’-ATTGGCCCAGGTCCCAG-3’ |
| Exon 8 | 5’-CAGGGCCCAGCATTAGAC-3 | 5’-CACCCTCTGATCCCTCCAG-3’ |
| Exon 9 | 5’-AGAAAATAGATTAAGTCCTTCCACTG-3’ | 5’-TGAACATTCCCACTGTGCTG-3’ |
| Exon 10 | 5’-CCCTGTTGTGGTTAGAAATGG-3’ | 5’-CGATGAAGCCAGAAGTCAGC-3’ |
| Exon 11 | 5’-ACTCAGGTCCTCCGGTCAC-3’ | 5’-AGCTTGGTGATGCCATGAG-3’ |
| Exon 12 | 5’-GTGTGGCAGATGGAGATGG-3’ | 5’-AGTGCTGCAGAATCAAAGGG-3’ |
| Exon 13 | 5’-TTGGTATATGAATGTGGAGCTATC-3’ | 5’-TTCCAGAAGGGTTTGGGAG-3’ |
