# Supplementary material for: Clinical and Functional Characterization of URAT1 Variants
Source: PLoS One. 2011 Dec 16;6(12):e28641. doi: 10.1371/journal.pone.0028641 (PMC3241677; doi:10.1371/journal.pone.0028641)
Supplement: Table S2 — Oligonucleotide primer pairs used for site-directed mutagenesis of SLC22A12. (DOC) [file pone.0028641.s002.doc]

**Table S2: Oligonucleotide primer pairs used for site-directed mutagenesis of SLC22A12**

|  | **Forward** | **Reverse** |
| --- | --- | --- |
| I75T | 5’-CTTGAGTCCTGAGGCCCTCCTGGCTACTTCCATCC-3’ | 5’-GGATGGAAGTAGCCAGGAGGGCCTCAGGACTCAAG-3’ |
| R347S | 5’-CCGCATGCCCGGACTGAGCTTCCGGACCTGTATC-3’ | 5’-GATACAGGTCCGGAAGCTCAGTCCGGGCATGCGG-3’ |
| V388M | 5’-GTTCATTGGTGTCATGGACATCCCAGC-3’ | 5’-GCTGGGATGTCCATGACACCAATGAAC-3’ |
| R434C | 5’-GAAATGGGGGCTCTGTGCTCAGCCTTGGCCGTG-3’ | 5’-CACGGCCAAGGCTGAGCACAGAGCCCCCATTTC-3’ |
| R434H | 5’-GAAATGGGGGCTCTGCACTCAGCCTTGGCCGTG-3’ | 5’-CACGGCCAAGGCTGAGTGCAGAGCCCCCATTTC-3’ |
